# Long-term Survivor of Intravascular Central Nervous System (CNS) Lymphoma: An Unusual Phenomenon

**DOI:** 10.1007/s12288-024-01948-y

**Published:** 2024-12-27

**Authors:** Amrita Guha, Jose Siju, Uma Sakhadeo, Tanuja Seth, Sachin Punatar, Jayant S. Goda

**Affiliations:** 1 Department of Radiodiagnosis, Advanced Centre for Treatment Research and Education in Cancer, Tata Memorial Centre, Kharghar, Navi Mumbai 410210 India; 2 Department of Pathology, Advanced Centre for Treatment Research and Education in Cancer, Tata Memorial Centre, Kharghar, Navi Mumbai 410210 India; 3 Department of Medical Oncology, Advanced Centre for Treatment Research and Education in Cancer, Tata Memorial Centre, Kharghar, Navi Mumbai 410210 India; 4 Department of Radiation Oncology, Advanced Centre for Treatment Research and Education in Cancer, Tata Memorial Centre, Kharghar, Navi Mumbai 410210 India; 5 Adult Hemato-Lymphoid Disease Management Group. Advanced Centre for Treatment Research and Education in Cancer, Tata Memorial Centre, Kharghar, Navi Mumbai 410210 India; 6 Homi Bhaba National Institute, Anushakti Nagar, Trombay, 400094 India; 7 Department of Radio-Diagnosis, Tata Memorial Hospital, Tata Memorial Centre, Parel, Mumbai, 400012 India

**Fig. 1:**
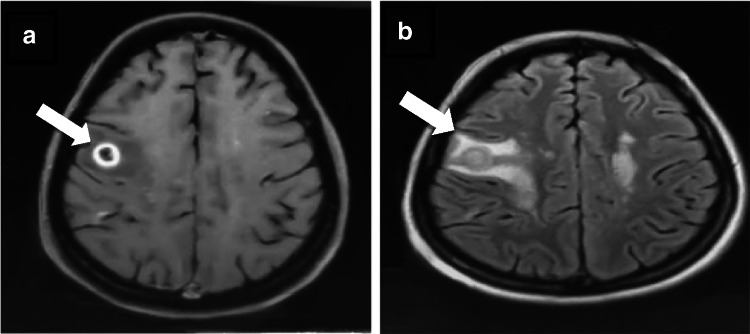
**a** Axial post-contrast T1W images showing ring enhancing lesion in the right parieto-frontal region. **b** Axial post-contrast T2/FLAIR images showing perilesional edematous change around the ring enhancing lesion

**Fig. 2:**
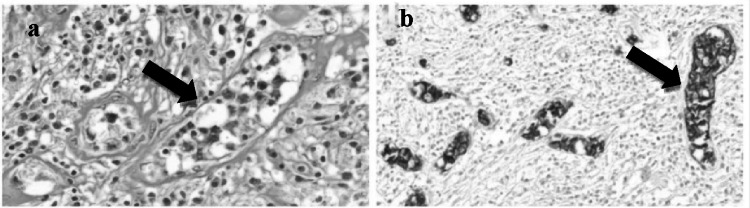
**a **× 400 magnification: Presence of large cells inside vascular lumina with surrounding perivascular edema. Intravascular large cells with conspicuous nucleoli admixed with leukocytes. **b **× 200 magnification. CD20 highlights the large cells confined to the vascular lumina

A 45-year-old Asian lady presented with history of fluctuating bilateral lower limb weakness for 2 months. MRI brain showed multiple ring and nodular enhancing lesions in the right fronto-parietal lobe (Fig. 1a, b). There was no systemic or cutaneous involvement. PETCT showed no extracranial disease. She underwent craniotomy and excision of lesion. Histopathological and immunohistochemistry revealed large lymphoid cells, predominantly in the vessels, strongly expressed CD-20, MUM1 and negative for CD3, CD10, BCL2 PDL1.MIB index was 80%. Neoplastic B-cells exclusively located in the blood vessel lumen (Fig. 2a, b) indicated a post-germinal centre Intravascular Large B-cell lymphoma (IVLBCL).

**Fig. 3:**
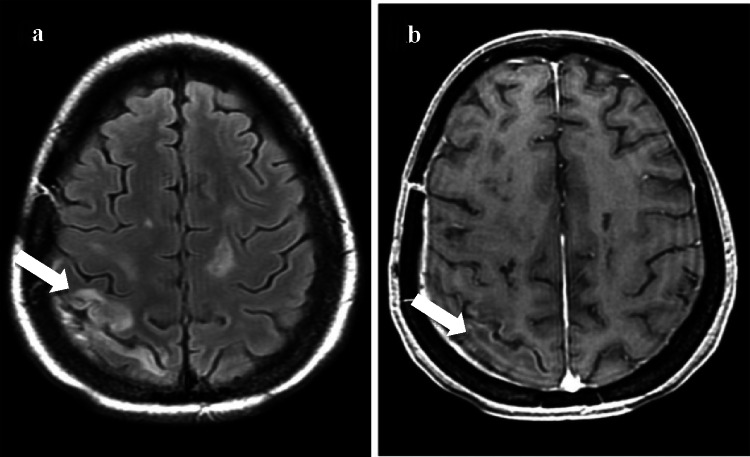
**a** Post-contrast T2/FLAIR imaging shows new onset altered signal intensity along the post-central gyrus 14 weeks after starting chemotherapy. **b** Post-contrast T1W image shows subtle leptomeningeal enhancement along post-central sulcus and superior parietal lobe

She was initiated on corticosteroids for symptomatic relief and treated with high-dose methotrexate and R-CHOP regimen. Follow-up MRI 14 weeks after initiation of treatment revealed new onset T2 intermediate signal in right post central gyrus and superior parietal lobule with leptomeningeal enhancement showing diffusion restriction, suggestive of disease progression (Fig. 3a, b). She received whole brain radiotherapy and conformal boost to the lesion followed by completion of RCHOP chemotherapy.

The patient is still alive at 3 years post-therapy albeit with residual focal neurological deficits. Follow-up MRI showed post-RT gliosis with no active lesions.

IVLBCL is rare, having an aggressive course and a median overall survival of less than a year with half of these patients showing CNS involvement [1]. It is characterized by an almost exclusive growth of large cells within the lumen of blood vessels. These lesions are a diagnostic dilemma due to their rarity and unconventional radiology. Unlike PCNSL, where lesions are solid T2 intermediate homogeneously enhancing, exhibit diffusion restriction, IVLBCL does not exhibit any pathognomonic imaging features on MRI. However, multiple T2/FLAIR hyperintensities, enhancement along perivascular spaces, and multifocal areas of diffusion restriction are common presentations that serve as potential clue for diagnosis [2].

Historically, IVLBCL has been considered to have dismal prognosis, however outcomes can be improved as illustrated in this case with rituximab and CNS directed therapy [1].
